# A Worldwide Survey of Activities and Practices in Clinical Islet of Langerhans Transplantation

**DOI:** 10.3389/ti.2022.10507

**Published:** 2022-08-11

**Authors:** Thierry Berney, Axel Andres, Melena D. Bellin, Eelco J. P. de Koning, Paul R. V. Johnson, Thomas W. H. Kay, Torbjörn Lundgren, Michael R. Rickels, Hanne Scholz, Peter G. Stock, Steve White, the International Islet Transplant Centers, Hamid R. Aghayan

**Affiliations:** ^1^ Division of Transplantation, Department of Surgery, University of Geneva Hospitals, Geneva, Switzerland; ^2^ Departments of Pediatrics and Surgery, University of Minnesota Medical Center, Minneapolis, MN, United States; ^3^ Department of Medicine, Leiden University Medical Center, Leiden, Netherlands; ^4^ Nuffield Department of Surgical Sciences, University of Oxford, John Radcliffe Hospital, Oxford, United Kingdom; ^5^ Department of Medicine, St. Vincent’s Hospital, St. Vincent’s Institute of Medical Research, University of Melbourne, Melbourne, VIC, Australia; ^6^ Department of Transplantation Surgery, Karolinska University Hospital, Stockholm, Sweden; ^7^ Division of Endocrinology, Diabetes and Metabolism, Department of Medicine, Institute for Diabetes, Obesity and Metabolism, University of Pennsylvania Perelman School of Medicine, Philadelphia, PA, United States; ^8^ Department of Transplant Medicine, Institute for Surgical Research, Oslo University Hospital, Oslo, Norway; ^9^ Division of Transplantation, Department of Surgery, University of California at San Francisco, San Francisco, CA, United States; ^10^ Department of HPB and Transplant Surgery, The Freeman Hospital, Newcastle Upon Tyne, United Kingdom

**Keywords:** islet transplantation, type 1 diabetes mellitus, activity, indications, health care coverage, ITA, IAK, SIK

## Abstract

A global online survey was administered to 69 islet transplantation programs, covering 84 centers and 5 networks. The survey addressed questions on program organization and activity in the 2000–2020 period, including impact on activity of national health care coverage policies. We obtained full data from 55 institutions or networks worldwide and basic activity data from 6 centers. Additional data were obtained from alternative sources. A total of 94 institutions and 5 networks was identified as having performed islet allotransplantation. 4,365 islet allotransplants (2,608 in Europe, 1,475 in North America, 135 in Asia, 119 in Oceania, 28 in South America) were reported in 2,170 patients in the survey period. From 15 centers active at the start of the study period, the number of simultaneously active islet centers peaked at 54, to progressively decrease to 26 having performed islet allotransplants in 2020. Notably, only 16 centers/networks have done >100 islet allotransplants in the survey period. Types of transplants performed differed notably between North America and the rest of the world, in particular with respect to the near-absence of simultaneous islet-kidney transplantation. Absence of heath care coverage has significantly hampered transplant activity in the past years and the COVID-19 pandemic in 2020.

## Graphical Abstract

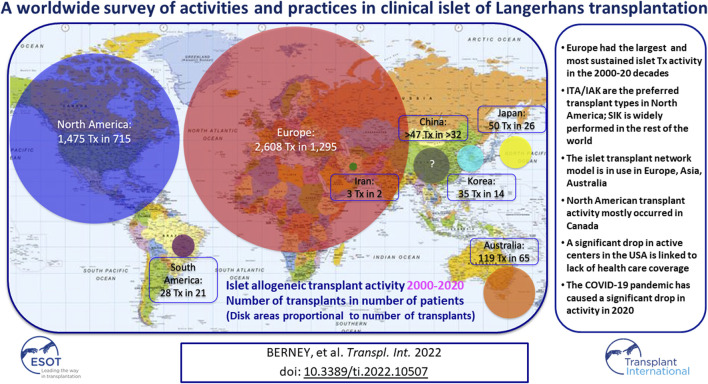

## Introduction

It has almost become commonplace to state that islet transplantation has become an established beta-cell replacement therapy since the seminal publication of the Edmonton protocol (1). The significant improvement of outcomes reported has led to a multiplication of islet transplant centers and transplant procedures. In comparison to the 237 procedures performed during the 1990–1999 decade and reported to the now defunct International Islet Transplant Registry (ITR) (2), 2,150 islet allotransplants have been reported to the Collaborative Islet Transplant Registry in the 1999–2015 period alone (3). The CITR is funded by the National Institute of Diabetes and Digestive and Kidney Diseases (NIDDK) and previously received support in part from the Juvenile Diabetes Research Foundation (JDRF). Therefore, collection, analysis, and communication of comprehensive and current data on human-to-human islet transplants is limited to those performed in transplant sites in North America, Europe and Australia, with NIDDK and JDRF sponsoring (3). As a result, the CITR data are skewed toward North American activity, with 1,146 procedures (53%) reported in Canada and the US alone, and the true number of islet transplant procedures performed worldwide is unknown.

The outcomes reported by the University of Alberta in 2000 have not only rekindled the interest in the procedure and boosted activity, but also led to a radical change in indication. Prior to the publication of the Edmonton protocol, simultaneous islet-kidney transplantation (SIK) was the most common indication (55%), followed by islet-after-kidney (IAK; 37%), islet transplant alone (ITA) being very rarely performed (4%) (2), and it is not exaggerated to say that, at least in North America, the Edmonton protocol has led to a true paradigm shift, with problematic hypoglycemia becoming the major indication for an islet transplant (4). However, CITR reports have provided hints that this change of practice may not have been as abrupt outside North America (3).

High-quality prospective clinical trials have been conducted in the past 2 decades and have demonstrated the value of islet transplantation in controlling complicated type 1 diabetes (5–8). Despite these achievements, islet transplantation still doesn’t benefit from third party health care coverage, most conspicuously, in the United States (9). Although this has not yet been studied, it is likely that reimbursement and activity should be correlated.

Finally, and more recently, the COVID pandemic has severely, albeit not in a uniform fashion, affected organ donation and transplantation activities globally (10). The impact on islet transplantation activity has not been studied.

The lack of actual activity data has prompted the authors to conduct an international survey with the aim to better characterize not only activity volumes, but also practices, including program organization, types of transplants performed, trends in activity and factors influencing those trends.

## Methods

### Survey Construction and Administration

In preparation for the American Diabetes Association’s 81st Scientific Sessions held virtually in 2021, the lead author (TB) of this study was tasked to give a lecture entitled “Successful implementation of clinical islet transplantation across the world: What can the US learn?”. A survey investigating worldwide islet transplant activity was designed to prepare for the lecture.

Survey was constructed and study data were collected and managed using the Microsoft Forms electronic data capture tool, hosted at the University of Geneva Hospitals. All centers, or at least one center per islet transplantation network, identified to have performed clinical islet allogeneic transplantation in the 2000–2020 period (11) received the questionnaire. Fifteen US centers who had terminated their allogeneic islet transplant activities (each of which had performed <10 transplants) could not be invited for lack of a contact. Questions were formulated to obtain information only on center practices and activity and included no request for outcome data. The survey included a combination of open and scroll-down menu questions. Questions were written to include “other” for all sections in order to allow for full description of alternative practices. The questionnaire is presented as Supplementary Appendix S1.

### Incomplete or Missing Data

Some datasets were completed, as per survey respondent instructions, with data obtained from the NHS-BT (UK National Health Service-Blood and Transplant) or ANZIPTR (Australia and New Zealand Islet and Pancreas Transplant Registry) activity reports available online (12, 13).

A minimal dataset (number and types of transplants performed on a yearly basis) was obtained from the CITR from North American institutions that had not been invited (N = 15) or had not responded to the survey (N = 5). For centers for which allotransplant numbers were obtained from the CITR, the number of transplanted patients was estimated, based on a theoretical ratio of 2 islet infusions for 1 recipient, as reported by North American centers to the CITR (infusion/recipient ratio: 1.95) (3). For these centers, activity was considered as “apparently terminated” if they had not performed/reported an islet transplant in >5 years.

A similar minimal dataset was obtained for 7 institutions in other continents who had terminated their islet transplant activities: from the Organizacion Nacional de Trasplantes of Spain (N = 3), from a survey administered in 2013 by the lead author of the present study (TB) and presented at the 14th World Congress of the International Pancreas and Islet Transplant Association (N = 3) and from a personal contact (N = 1) (14).

For 4 non-responding centers, all located in China, no current data could be retrieved. Data on patients transplanted in 2 centers in China were added to the activity calculation as obtained from the 2013 survey mentioned above for 1 center and from a publication for the other (15).

### Data Analysis

Data are presented and analyzed for each individual center and for each network or consortium. Descriptive statistics were performed using Microsoft Forms and Excel.

## Results

### Response to the Survey

Invitations to take the survey were sent to 69 program directors, covering 84 centers/5 networks worldwide. We received a response to the survey from 55 (79.7%), covering 65 centers and 5 networks. We obtained partial responses from 6 additional centers (4 terminated, 2 with only an autotransplant program), for a total response rate to the survey of 88.4%.

A list of institutions and networks, with survey response and source of data details is provided in the Supplementary Table.

### Islet Transplant Centers and Networks

**FIGURE 1:**
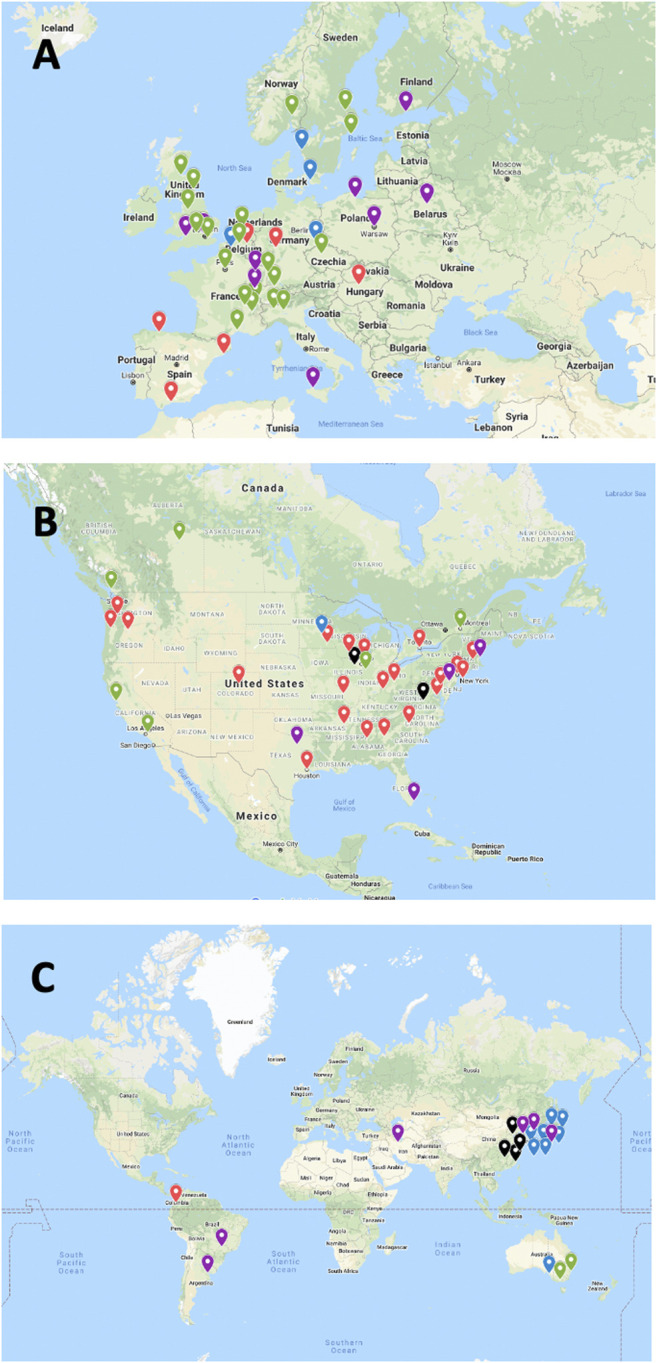
Geographic location and activity status of 94 institutions performing or having performed allogeneic islet transplantation (2000–2020). **(A)** Europe; **(B)** North America; **(C)** “Rest of the World”. Green marker: active centers; Blue marker: active centers without activity in 2020; Purple marker: activity on hold; Red marker: activity terminated; Black marker: current status unknown.

After integration of all data obtained as indicated above, 103 islet transplant centers were identified, of which 94, in 25 countries, had reported allotransplantation activity during the survey period. Fifteen islet allotransplant centers in 4 countries are located in Asia (16%), 39 in 15 countries in Europe (42%), 34 in 2 countries in North America (36%), 3 in 1 country in Oceania (3%) and 3 in 3 countries in South America (3%). Their geographic location is presented in Figure 1.

For 85 of 94 centers with relevant data, 45 had a combined allo- and auto-transplant program (53%) and 40 an allo-program only (47%). These proportions varied significantly between North America, Europe and other continents (Supplementary Figure S1).

A small majority of programs integrate an islet isolation facility and a local islet transplantation program (35/67; 52%). Thirty islet transplant centers (45%) are organized in 5 networks, built around 17 islet isolation facilities, with different functioning modalities. Table 1 summarizes the list of networks. Two additional transplant centers (activity terminated) have transplanted islets shipped from another institution in bilateral collaborations (Houston/Miami; Budapest/Geneva).

**TABLE 1:** Islet transplantation networks.

Network	Countries	Number of isolation facilities	Number of transplant centers	Shipment of islets
Japan Islet Transplant Consortium Consortium	Japan	7	7	No
GRAGIL Network	France Switzerland	2	7	Yes
Nordic Network for Clinical Islet Transplantation	Denmark Finland Norway Sweden	2	6	Yes
UK Islet Transplant Consortium (UKITC)	United Kingdom	4^a^	7^b^	Yes
Australian Islet Consortium	Australia	2	3	Yes

a One facility currently on hold or terminated.

b Two transplant centers (including one with facility) currently on hold.

A list of institutions and networks, with allogeneic and autologous transplantation details, and current activity status is provided in the Supplementary Table.

### Islet Transplant Activity

Between January 2000 and December 2020, 4,321 islet allotransplants in 2,149 patients were reported worldwide. Islet transplant products pooled from 2 or more islet preparations were counted as a single islet transplant. Most islet transplants were performed in Europe (2,608, 59.7%), followed by North America (1,475, 33.8%), Asia (135, 3.1%), Oceania (119, 2.7%) and South America (28, 0.6%).

**FIGURE 2:**
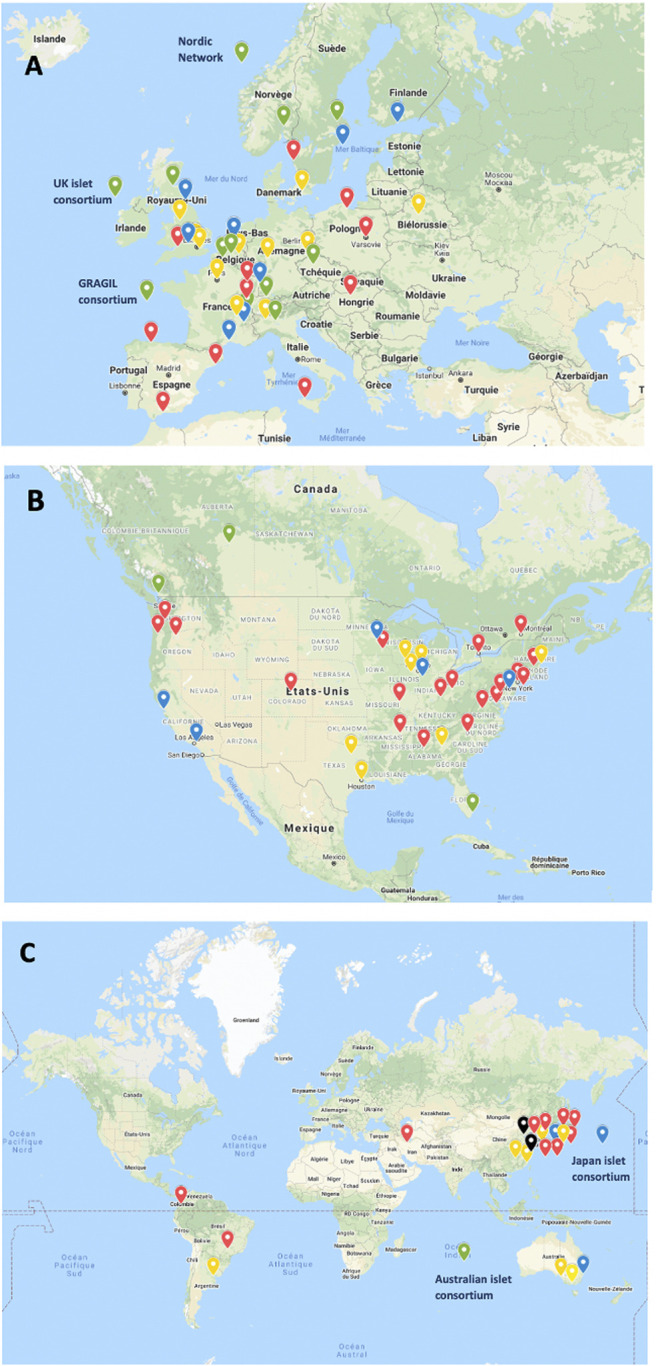
Islet allotransplant activity of 94 institutions/networks performing or having performed islet transplantation (2000–2020). **(A)** Europe; **(B)** North America; **(C)** “Rest of the World”. Green marker: ≥100 transplants performed; Blue marker: 50–99 transplants performed; Yellow marker: 10–49 transplants performed; Red marker: <10 transplants performed.

**FIGURE 3:**
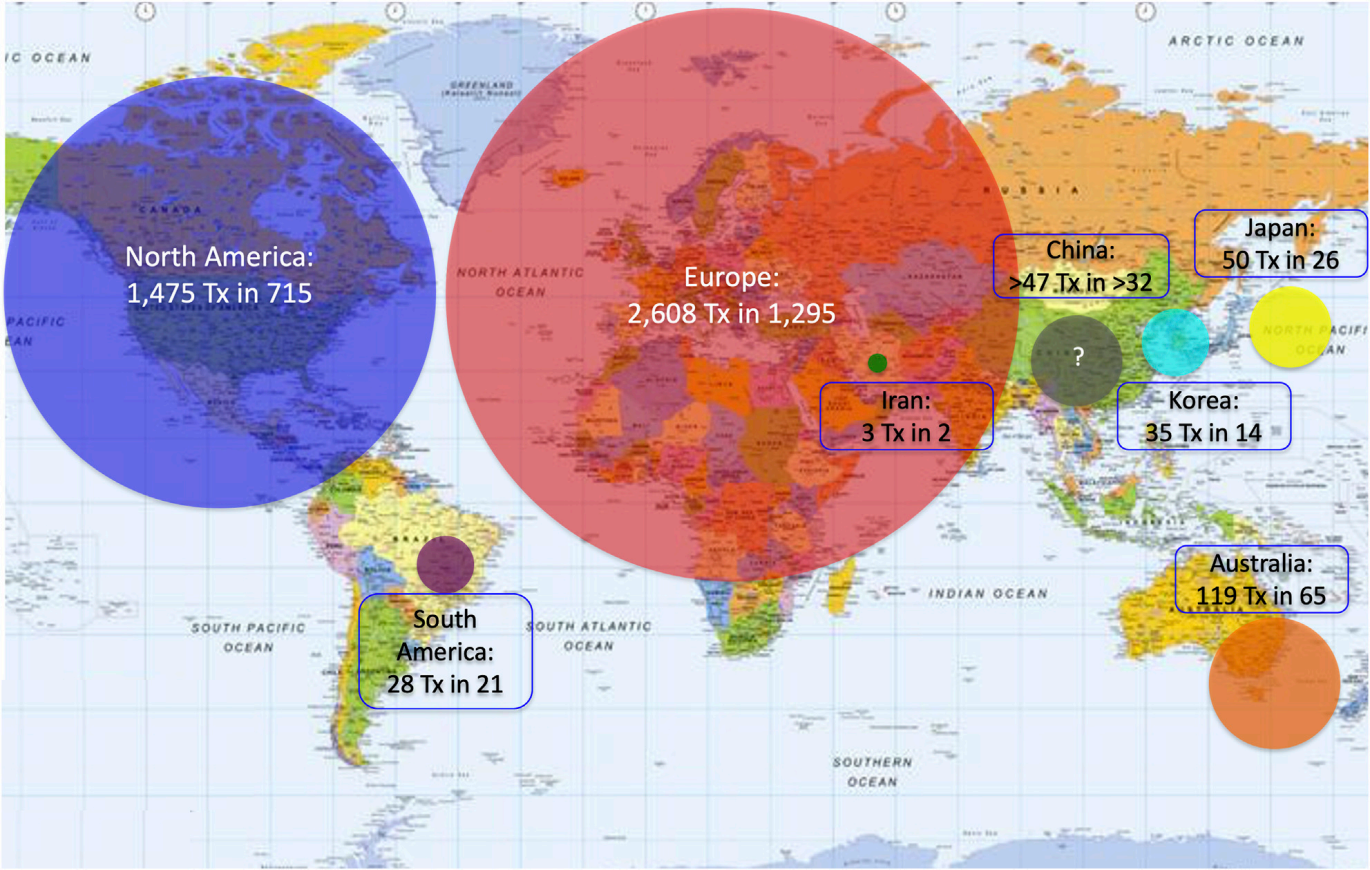
Continental/regional distribution of islet allotransplant activities (2000–2020). Surface of color disks is proportional to number of transplants performed.

A great variation in the levels of activity was observed, 41 centers (44%) having reported <10 transplants and only 12 having (13%) reported ≥100 transplants. Four of 5 islet transplant networks have reported ≥100 transplants. Center-specific activity appears in the Supplementary Table. The geographic location of centers or networks according to total activity appears in Figure 2 and centers and networks having performed ≥100 transplants are listed in Table 2. The continental distribution of transplant activities is summarized in Figure 3.

**TABLE 2:** Islet transplantation centers or networks having reported ≥100 islet allotransplants^a^.

Center/Network	Countries	Number of transplants	Number of patients
University of Alberta—Edmonton	Canada	681	293
Nordic Network for Clinical Islet Transplantation	Denmark Finland Norway Sweden	458	199
GRAGIL Network	France Switzerland	457	234
UK Islet Transplant Consortium (UKITC)	UK	331	189
Brussels Free University	Belgium	273	102
Geneva University^b^	Switzerland	205	115
Lille University	France	171	61
University of Uppsala^c^	Sweden	167	60
San Raffaele Institute—Milan	Italy	162	87
University of British Columbia—Vancouver	Canada	142	60
University of Oslo^c^	Norway	127	48
Zurich University	Switzerland	120	54
Australian Islet Consortium	Australia	119	65
IKEM _ Prague	Czech Republic	114	68
Edinburgh Royal Infirmary^d^	UK	113	60
University of Miami	United States	100	56

a Number of islet infusions is counted, regardless of number of islet preparations pooled.

b Also included in “GRAGIL Network” numbers.

c Also included in “Nordic Network for Clinical Islet Transplantation” numbers.

d Also included in “UK Islet Transplant Consortium” numbers.

### Period of Activity

**FIGURE 4:**
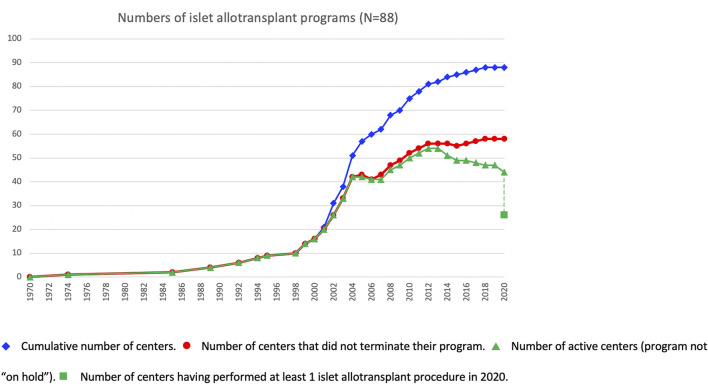
Evolution over time of islet transplant center activities. 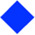 Cumulative number of centers. 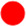 Number of centers that did not terminate their program. 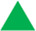 Number of active centers (program not “on hold”). 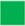 Number of centers having performed at least 1 islet allotransplant procedure in 2020.

At the beginning of the survey period, 16 of the active centers had performed at least 1 islet allotransplant procedure before 1 January 2000, including 10 in Europe (Milan-San Raffaele, Giessen, Oxford, Brussels-VUB, Geneva, Lille, Grenoble, Strasbourg, Lyon, Besançon, Stockholm), 3 in North America (Minneapolis, Miami, Edmonton), 1 in Asia (Seoul-Samsung) and 1 in South America (Buenos Aires). Additional centers had started and discontinued allogeneic islet transplant programs in the 1990s (2), only one of which (Saint-Louis) resumed its activities in the study period. The number of active islet transplant centers changed continuously during the period, new centers opening and active centers terminating or putting their activity on hold. The evolution over time of the number of active islet centers is presented in Figure 4. Of 88 centers with available date, 30 terminated (reportedly or apparently) their activity, and an additional 15 have put their activity on hold. Forty-four centers reported as being active at the time of survey, of which only 26 have performed at least 1 transplant in 2020. Overall, 47% of centers are still reportedly active worldwide, 20% have reportedly put their islet allotransplant program “on hold” and 33% have reportedly or apparently terminated their programs. These proportions vary significantly between world regions (Supplementary Figure S2), with a much higher percentage of terminated programs in North America (66%) than in Europe (15%) or the rest of the world (6%).

### Types of Transplants Performed

Of 65 centers with relevant data, islet transplantation was performed as islet-after-kidney (IAK) by 89%, islet transplant alone (ITA) by 83% and simultaneous islet-kidney (SIK) by 32%.

IAK is performed in 100%, 82% and 67%; ITA in 80%, 100% and 67%; and SIK in 55%, 13% and 25%, of centers from Europe, North America and the rest of the world, respectively.

To the question about the preferred procedure performed (several answers possible), 44% replied ITA, 24% both ITA and IAK, 16% IAK and 16% SIK, either alone or in combination with IAK, ITA or both, without major continental differences.

Of 5 networks, 2 (Japan, Nordic) perform both ITA and IAK as their only and preferred procedures. In the remaining 3, ITA, IAK and SIK have been performed, but practices vary from center to center.

**FIGURE 5:**
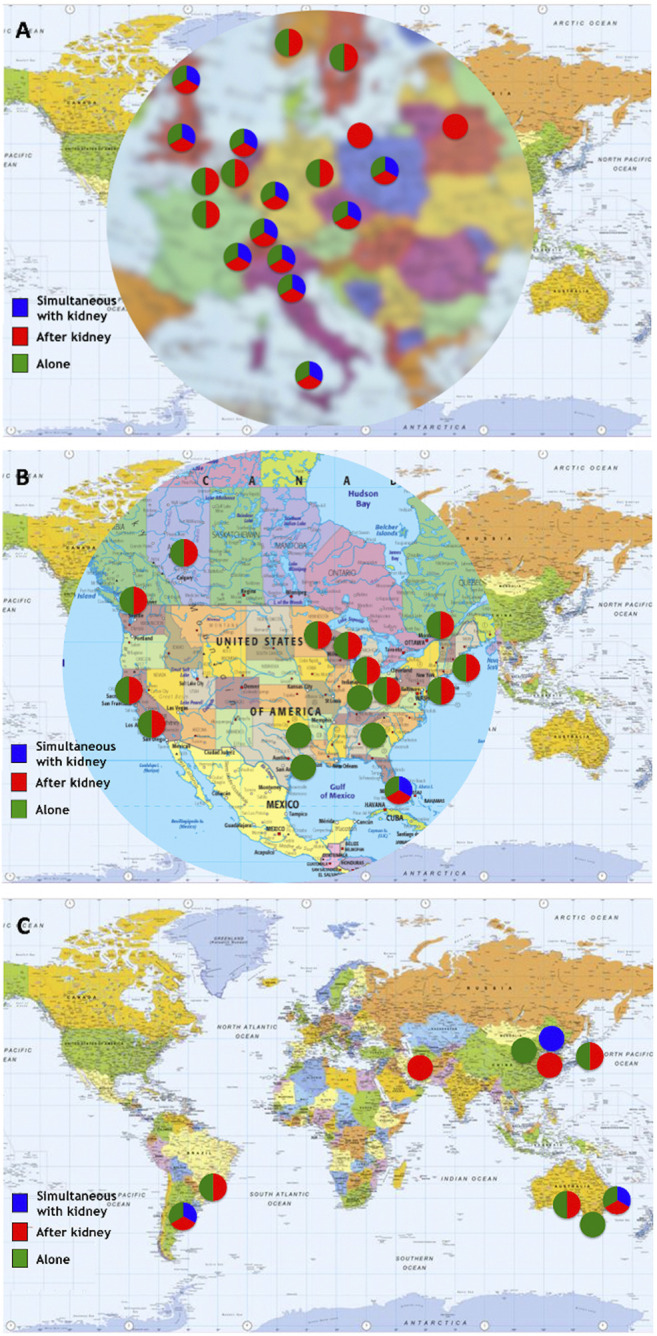
Types of transplants performed by center/network. **(A)** Europe; **(B)** North America; **(C)** “Rest of the World”.

**FIGURE 6:**
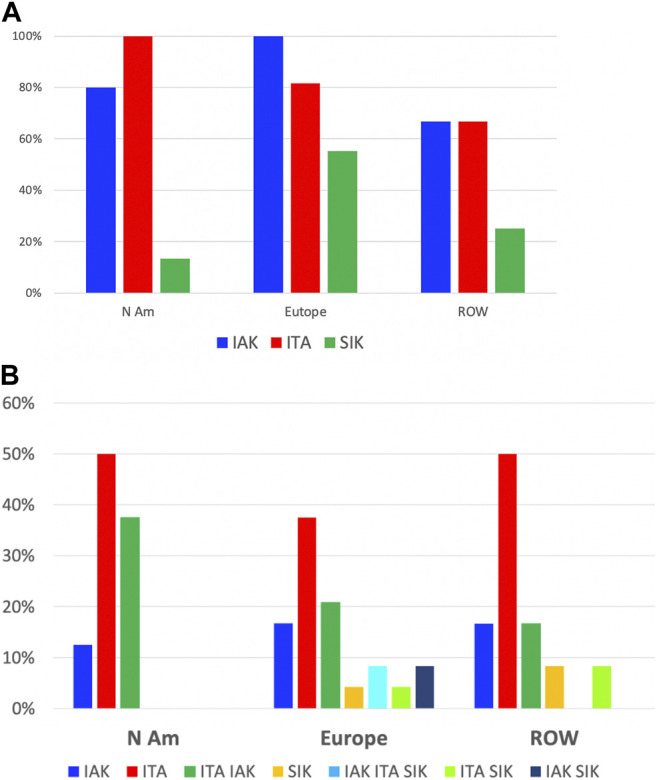
Types of transplants performed: continental distribution. **(A)** Proportion of centers in each region having performed IAK (blue), ITA (red) or SIK (green). **(B)** Proportion of centers in each region by overall preferred types of transplants; blue: IAK only, red: ITA only, green: ITA and IAK, ochre: SIK only, turquoise: IAK, ITA and SIK, light green: ITA and SIK, black: IAK and SIK.

Eight centers (6 in Europe, 2 in North America) reported performance of islet allotransplantation in other combinations, namely simultaneous islet-lung or islet-after-lung in cystic fibrosis patients (16, 17), simultaneous islet-liver (including simultaneous islet-lung-liver (18) and simultaneous islet-liver-kidney (19)) and simultaneous islet-heart transplantations. The geographic and continental distribution of types of transplant performed are summarized in Figures 5, 6. The center-by-center distribution appears in the Supplementary Table.

Fourteen centers have reported performing islet allogeneic transplantation in extra-hepatic sites (20), including 9 in the omentum (21, 22), 4 in the skeletal muscle (23), 3 in the gastric submucosa (24), 2 inside devices (25), 1 in the bone marrow (26) and 1 in the anterior chamber of the eye.

### Internal Organization

Allogeneic islet transplant programs (data from 67 centers and 5 networks) are or were directed in majority by surgeons (30 centers, 2 networks; 44%) or diabetes/endocrinology (D/E) specialists (25 centers, 1 network; 37%). The remainder being led by nephrologists (7 centers, 1 network; 10%), a joint team of a D/E specialist and a surgeon (4 centers; 6%), a joint team of surgeon and immunologist (1 network), a joint team of nephrologist and D/E specialist (1 center) or a joint team of lab director and nephrologist (1 center). Program leadership differed significantly between North America (62% surgeons; 24% D/E), Europe (38% D/E; 31% surgeons) and other continents (43% D/E; 36% surgeons).

We obtained data on the co-existence of a pancreas transplant program in the same institution from 64 respondents. Forty-eight institutions had an islet and a pancreas transplant program (75%) and 16 only had an islet transplant program (25%). Of 40 centers with available data, the pancreas and islet transplant programs were described as a joint program in 17 (43%), as separate programs with close interaction in 20 (50%), 2 centers reported separate programs with occasional interaction and 1 center indicated totally separate programs. There were no major continental differences in these organizations.

In 20 institutions reporting a joint program, 16/75%) see their referrals in the same initial visit and discuss them in the same WL conference. For the remainder, referrals are directed to different visits in 2 instances and discussed in separate waiting list (WL) conferences in 2 instances.

Programs described as separate but with close interactions had a similar organization, with a single referral visit and WL conference in 67%. Interestingly, one center described as separate programs with occasional interaction has a unique referral visit, but separate waiting lists. As expected, the remainder had separate referral visits and WL conference.

Of 20 countries in which at least one center responded, 17 (85%) have 2 separate national WLs for islet and pancreas and only 3 (15%) have a single WL (Belarus, Switzerland, United Kingdom).

### Health Care Coverage for Islet Allotransplantation

Allogeneic islet transplantation is fully covered by the health care system in 9 countries, namely Australia, Belarus, Canada (Alberta, British Columbia), Finland, France, Iran, Poland, Sweden, Switzerland and the United Kingdom. Reimbursement was reported as partial in Belgium, the Czech Republic, Germany, Italy, Japan, and Norway. The procedure for securing health care coverage was reported as initiated in one further province of Canada (Quebec), but coverage was obtained in the few weeks preceding submission of this report. Islet allotransplantation was reported as not reimbursed, but under evaluation in the Netherlands, and as not reimbursed with no clear perspective in Argentina, Brazil and Korea. Interestingly, for the United States, where islet allotransplantation is not covered, 7/12 survey respondents evaluated the situation as “not reimbursed with no clear perspective”, and 5 as “under evaluation” (9).

### Impact of COVID Pandemic and Other Factors on Islet Transplant Activity

Of 20 centers who reported a terminated or temporarily on hold program, reasons were regulatory/lack of health care coverage for 6 (4 in the United States, 1 in Korea, 1 in Argentina), logistic for 3, COVID pandemic for 3, institutional decision for 1, and financial unrelated to regulations for 1; six centers did not indicate a reason.

The impact of the COVID pandemic could be assessed for 29 reportedly active centers and 3 temporarily on hold for COVID reasons. Overall, 13 reported a decrease in activity and 13 a temporary or ongoing interruption of their program. Six active centers reported an absence of impact, of which only 3 performed at least 1 allotransplant in 2020. The geographical distribution of these centers appears in the Supplementary Figure S3.

## Discussion

This study has the merit to present a comprehensive picture of the worldwide allogeneic islet transplant activity in the past 2 decades, i.e., with a starting time point represented by the publication of the seminal Edmonton study (1). It does not only reveal raw activity numbers, but also unveils certain differences between North America—mainly the US- and other continental regions of the world in terms of indications, organization and practices. The major strength of the study is the high rate of response to the survey. One limitation is that it does not provide outcome data, a deliberate choice made by the authors to ensure a maximal response to the survey.

Despite the limitations inherent in a survey, all transplant numbers are accurately reported for almost every country in the world, with the exception of the United States and China. Activity numbers of US centers who did not reply to the survey or could not be contacted were obtained from the CITR, which captures nearly all allogeneic islet transplant activity in the US, which has remained dependent on NIH and JDRF support of clinical trials, with mandatory reporting in the absence of biologic licensure. It should be mentioned here that a certain level of underreporting is expected, but is likely to be minimal, especially since most of these centers had terminated their activity, which included <10 transplants in all but 2 institutions. For China, none of the 4 institutions contacted replied to the survey. We obtained information on transplants performed from a 2013 publication (15) or from a survey previously performed in 2013 (see Methods), for one center each, but it is unknown whether these centers have an ongoing allogeneic islet transplant activity or not. Therefore, Chinese data are an underestimation based on partial data. Finally, islet transplant centers were identified based on current and previously existing registries (2, 3, 12, 13), literature searches (11) and personal connections. It is therefore possible that not all active centers were really identified, but missing institutions are unlikely to have significantly contributed in terms of activity volumes.

The survey allowed to identify 94 programs having performed allogeneic islet transplantation in the study period. The decision to start the survey study period in 2000 was arbitrarily chosen because it coincided with the publication of the seminal “Edmonton protocol” paper, which was widely considered to be a game changer in the field at the time (1). The success of the “Edmonton protocol” was not so much a quantum leap as a particular step -albeit a significant one-in a history of continuous progress, from the first demonstration of diabetes reversal in rodent experiments by Paul Lacy in Saint-Louis, to the first clinical islet transplants by D. Sutherland and J. Najarian, the invention of the automated method of islet isolation by Camillo Ricordi and further advances in Europe and North America (27).

Of the 94 programs active in these 2 decades, only two pioneering institutions had performed >10 procedures, namely the University of Pittsburgh (26 procedures), who eventually elected to focus on autotransplantation, and Washington University in Saint Louis, who resumed an allogeneic program in 2000 (2). Several institutions active in the field in the 1990s had terminated their activities, at least temporarily by the start of the study period (2).

Looking at transplant figures, the good news is that there has been a steady increase in activity. In a previous survey-based report presented at the 2013 IPITA congress, 2,349 transplants in 1,178 patients had been reported, as compared to 4,322 in 2,150 in the present study, i.e., a near-doubling in 7 years.

The improvement of islet transplant outcomes reported by the Edmonton protocol has somewhat overshadowed the paradigm shift represented by the focus on problematic hypoglycemia and ITA as the foremost indication. Indeed, in the 1990s, ITA represented <5% of all islet transplant procedures, and SIK >50% (2). The Edmonton protocol has been much more impactful in North America than in the rest of the world, where IAK and SIK have been much more commonly performed. In this regard, it should be pointed out that technology has been improving exponentially as islet programs have been progressing. The sole hypoglycaemia unawareness indication for ITA may have led to a reduction in referrals as sensor-augmented pumps tend to become the norm (4). Broader indications and inclusion criteria have therefore been explored and implemented.

Another notable difference is the organization in islet transplant consortia, albeit with variations in the types and levels of interaction, that has been embraced in Europe, Australia and Japan, but not in North America. National, or even regional (GRAGIL, Nordic Network), networks, with transplant centers located around a centralized islet production laboratory, facilitate access to islet transplantation and ease the burden of traveling to a distant islet center for the patients. These models undoubtedly have a positive impact on finances and equity of access (28), and national health policymakers should consider promoting and implementing their construction in countries where they do not exist.

In the survey period, allogeneic islet transplantation activity has been mostly performed in Europe, and the differences have accentuated in the past 7 years. High activity levels have progressively shifted from North America to Europe, both in terms of patients transplanted, but also active islet transplant centers. It is striking that more than half of North American transplants have been performed in Canada (833 transplants in 360 patients, versus 642 in 255 for the United States). This is an unsurprising and expected result of the regulatory framework in the United States, in which allogeneic islets are considered a biologic drug, with the ensuing difficulties met by academic institutions to comply with the tremendous logistic and financial consequences (9, 29). This situation seems to be unique to the US, in contrast to Canada and most countries in the rest of the world, where allogeneic islets are considered as cell therapy products and fall under organ transplant regulations (30, 31). The US regulation implies that a Biologics License must be obtained for an institution or a company to be authorized to manufacture and administer allogeneic islets to patients with type 1 diabetes and to secure third party reimbursement. No such license has been granted so far, resulting in the absence of health care coverage in the United States. Many other countries currently have islet transplantation considered as standard-of-care and reimbursed, but there are still several instances in which the classification of allogeneic islets as “basic” cell therapy products has not led to their recognition as standard-of-care treatment of type 1 diabetes and/or to full or even partial insurance coverage.

Over the study period, new centers have constantly opened, and established centers terminated, or put on temporary hold, their activities. Overall, after a regular net increase in the first decade of the century, a net drop in the number of active centers, from a peak of 56 in 2012, can be observed in the past decade. As shown by the data, this is mostly due to the closure of US centers for the reasons outlined above. An interesting point is the important shift of practice adopted by US centers in response to this deadlock situation, and a majority have focused their activity on islet autotransplantation programs.

The impact of the COVID pandemic cannot be overestimated. Activity has decreased or even been interrupted because of the COVID situation in a vast majority of reporting centers, and is accountable for a 40% drop in transplant activity (in terms of active centers) in 2020 with respect to the previous year.

This comprehensive survey was able to quantify islet transplant allotransplantation in the past 2 decades and to identify differences in activity and practices in different regions of the world. Although a steady activity has been reported over the study period, absence of heath care coverage and the COVID-19 pandemic have significantly hampered transplant activity in 2020. This survey gives a rather accurate description of the activity in a critical period in time, but is only a snapshot, that cannot replace data from a comprehensive worldwide registry, unfortunately unavailable at this time. Although the CITR is an extraordinary source of valuable data, this survey indicates that it does not capture a major part of the international islet transplant activities and outcomes (6 of the 16 centers or networks having performed >100 transplants do not report to CITR). The ANZIPTR and NHS-BT registry are publicly available registries containing a wealth of data on islet and pancreas transplantation in Australia/New Zealand, respectively the UK, including outcomes (12, 13). The European Pancreas and Islet Transplant Registry (EPITR) is a current effort from ESOT/EPITA aiming at covering these needs for Europe (32). Similar coordinated efforts should be made in other parts of the world and integrated into a truly international islet transplant registry capturing all the activity.

## The Members of the International Islet Transplant Centers

Hamid R. Aghayan (Tehran University of Medical Sciences, Tehran, Iran); Rodolfo Alejandro (University of Miami, Miami, FL, United States); Takayuki Anazawa (Kyoto University, Kyoto, Japan); Axel Andres (University of Geneva, Geneva, Switzerland); Mathieu Armanet (Hôpital Saint-Louis, Paris, France); Lionel Badet (Hospices Civils de Lyon, Lyon, France); Appakalai N. Balamurugan (University of Louisville, Louisville, KY, United States); Franca B. Barton (Collaborative Islet transplant Registry, Rockville, MD, United States); Melena D. Bellin (University of Minnesota, Minneapolis, MN, United States); Pierre Y. Benhamou (Grenoble-Alpes University, Grenoble, France); Ekaterine Berishvili (University of Geneva, Geneva, Switzerland); Thierry Berney (University of Geneva, Geneva, Switzerland); Federico Bertuzzi (Niguarda Hospital, Milan, Italy); Sophie Borot (Université de Franche-Comté, Besançon, France); Domenico Bosco (University of Geneva, Geneva, Switzerland); Rita Bottino (University of Pittsburgh, Pittsburgh, PA, United States); Mathias D. Brendel (Justus-Liebig University, Giessen, Germany); Fanny Buron (Hospices Civils de Lyon, Lyon, France); Luis Armando Caicedo (Fundacion Valle Del Lili, Cali, Colombia); John Casey (Royal Infirmary of Edinburgh, Edinburgh, United Kingdom); Pierre Cattan (Hôpital Saint-Louis, Paris, France); Pratik Choudhary (King’s College, London, United Kingdom); Patrick T. Coates (Royal Adelaide Hospital, Adelaide , Australia); Ignacio Conget (Hospital Clinic, Barcelona, Spain); Matthew Cooper (Medstar Georgetown Transplant Institute, Washington, DC, United States); Eelco J.P. de Koning (University of Leiden, Leiden, The Netherlands); Alicja Dębska-Ślizień (Medical University of Gdansk, Gdansk, Poland); Chirag S. Desai (University of North Carolina, Chapel Hill, NC, United States); Beatriz Dominguez-Gil (Organizacion Nacional de Trasplantes, Madrid, Spain); Julian Drago (Hospital Italiano, Buenos Aires, Argentina); ^‡^Gabriel J. Echeverri (Fundacion Valle Del Lili, Cali, Colombia); Dzmitry Fedaruk (Republican Center for Organ and Tissue Transplantation, Minsk, Belarus); Justyna Gołębiewska (Medical University of Gdansk, Gdansk, Poland); John A. Goss (Baylor College of Medicine, Houston, TX, United States); Bernhard J. Hering (University of Minnesota, Minneapolis, MN, United States); Sung H. Hyon (Hospital Italiano, Buenos Aires, Argentina); Paul R.V. Johnson (University of Oxford, Oxford, United Kingdom); Hye S. Jung (Seoul National University Hospital, Seoul, Korea); Mazhar A. Kanak (Virginia Commonwealth University, Richmond, VA, United States); Fouad Kandeel (City of Hope Medical Center, Duarte, CA, United States); Thomas Kay (Saint Vincent Institute, Melbourne, Australia); Takashi Kenmochi (Japan Islet Transplant Registry, Toyoake, Japan); Laurence Kessler (Strasbourg University, Strasbourg, France); Bart Keymeulen (Brussels Free University, Brussels, Belgium); Hussein Khambalia (University of Manchester, Manchester, United Kingdom); Khalid M. Khan (Medstar Georgetown Transplant Institute, Washington, DC, United States); Jae H. Kim (Samsung Medical Center, Seoul, Korea); Olle Korsgren (University of Uppsala, Uppsala, Sweden); Yogish C. Kudva (Mayo Clinic, Rochester, MN, United States); Sandrine Lablanche (Grenoble-Alpes University, Grenoble, France); Leticia Labriola (University of Sao Paulo, Sao Paulo, Brazil); Eun Y. Lee (Seoul Catholic University, Seoul, Korea); Roger Lehmann (University of Zurich, Zurich, Switzerland); Barbara Ludwig (Technische Universität Dresden, Dresden, Germany); Törbjörn Lundren (Karolinska Institute, Stockholm, Sweden); Xunrong Luo (Northwestern University, Chicago, IL, United States); Paola Maffi (San Raffaele Institute, Milan, Italy); James Markmann (Harvard University, Boston, MA, United States); Alessandro Mattina (IRCCS ISMETT – UPMC Italy, Palermo, Italy); Mark Meloche (University of British Columbia, Vancouver, Canada); Sri Prakash L. Mokshagundam (University of Louisville, Louisville, KY, United States); Katherine Morgan (Medical University of South Carolina, Charleston, SC, United States); Marion Munch (Strasbourg University, Strasbourg, France); Bashoo Naziruddin (Baylor Simmons Transplant Institute, Dallas, TX, United States); Jon S. Odorico (University of Wisconsin, Madison, WI, United States); Nicholas Onaca (Baylor Simmons Transplant Institute, Dallas, TX, United States); Steven Paraskevas (McGill University, Montreal, Canada); François Pattou (Lille University, Lille, France); Nadine Pernin (University of Geneva, Geneva, Switzerland); Lorenzo Piemonti (San Raffaele Institute, Milan, Italy); Michael R. Rickels (University of Pennsylvania, Philadelphia, PA, United States); Camillo Ricordi (University of Miami, Miami, FL, United States); Raul Muñoz Romo (Organizacion Nacional de Trasplantes, Madrid, Spain); Frantisek Saudek (Institute for Clinical and Experimental Medicine, Prague, Czech Republic); Hanne Scholz (Oslo University, Oslo, Norway); James A.M. Shapiro (University of Alberta, Edmonton, Canada); James Shaw (Freeman Hospital, Newcastle-upon-Tyne, United Kingdom); Masayuki Shimoda (National Center for Global Health and Medicine, Tokyo, Japan); Mark D. Stegall (Mayo Clinic, Rochester, MN, United States); Peter G. Stock (University of California (UCSF), San Francisco, CA, United States); Marie-Christine Vantyghem (Lille University, Lille, France); Pedro Ventura-Aguiar (Hospital Clinic, Barcelona, Spain); Charles H. Wassmer (University of Geneva, Geneva, Switzerland); Angela Webster (University of Sydney, Sydney, Australia); Steve White (Freeman Hospital, Newcastle-upon-Tyne, United Kingdom); Piotr Witkowski (University of Chicago, Chicago, IL, United States); Anne Wojtusciszyn (Montpellier University, Montpellier, France); Michal Wszola (Foundation of Research and Science Development, Warsaw, Poland); Kun-Ho Yoon (Seoul Catholic University, Seoul, Korea).

## Data Availability

The original contributions presented in the study are included in the article/Supplementary Material, further inquiries can be directed to the corresponding author.

## Footnotes

‡ Deceased.

## Supplementary Material

Supplementary Figure S1 Proportions of islet allotransplant centers harbouring an autotransplant program.

Supplementary Figure S2 Proportions of islet allotransplant centers according to current activity status.

Supplementary Figure S3 Impact of the COVID-19 pandemic on islet allotransplantation activity. **(A)** Europe; **(B)** North America; **(C)** “Rest of the World”.

Supplementary Table S1 List of islet transplant centers.

Supplementary Datasheet S1 Appendix: Survey form.
