# Supplementary figures and images for: A Worldwide Survey of Activities and Practices in Clinical Islet of Langerhans Transplantation
Source: Transpl Int. 2022 Aug 11;35:10507. doi: 10.3389/ti.2022.10507 (PMC9402897; doi:10.3389/ti.2022.10507)

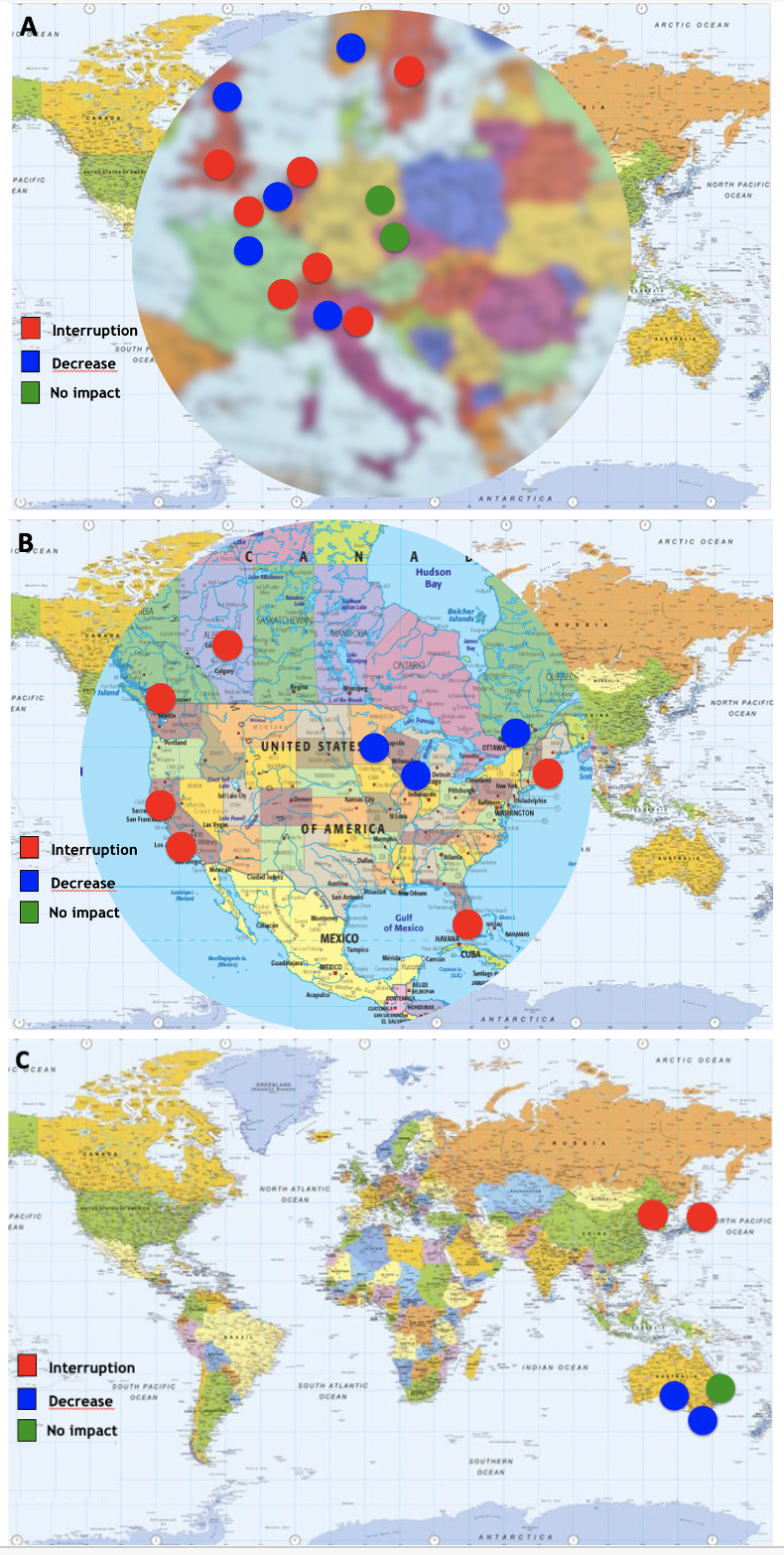

Supplement: Supplementary file 1 [file Image3.TIFF]

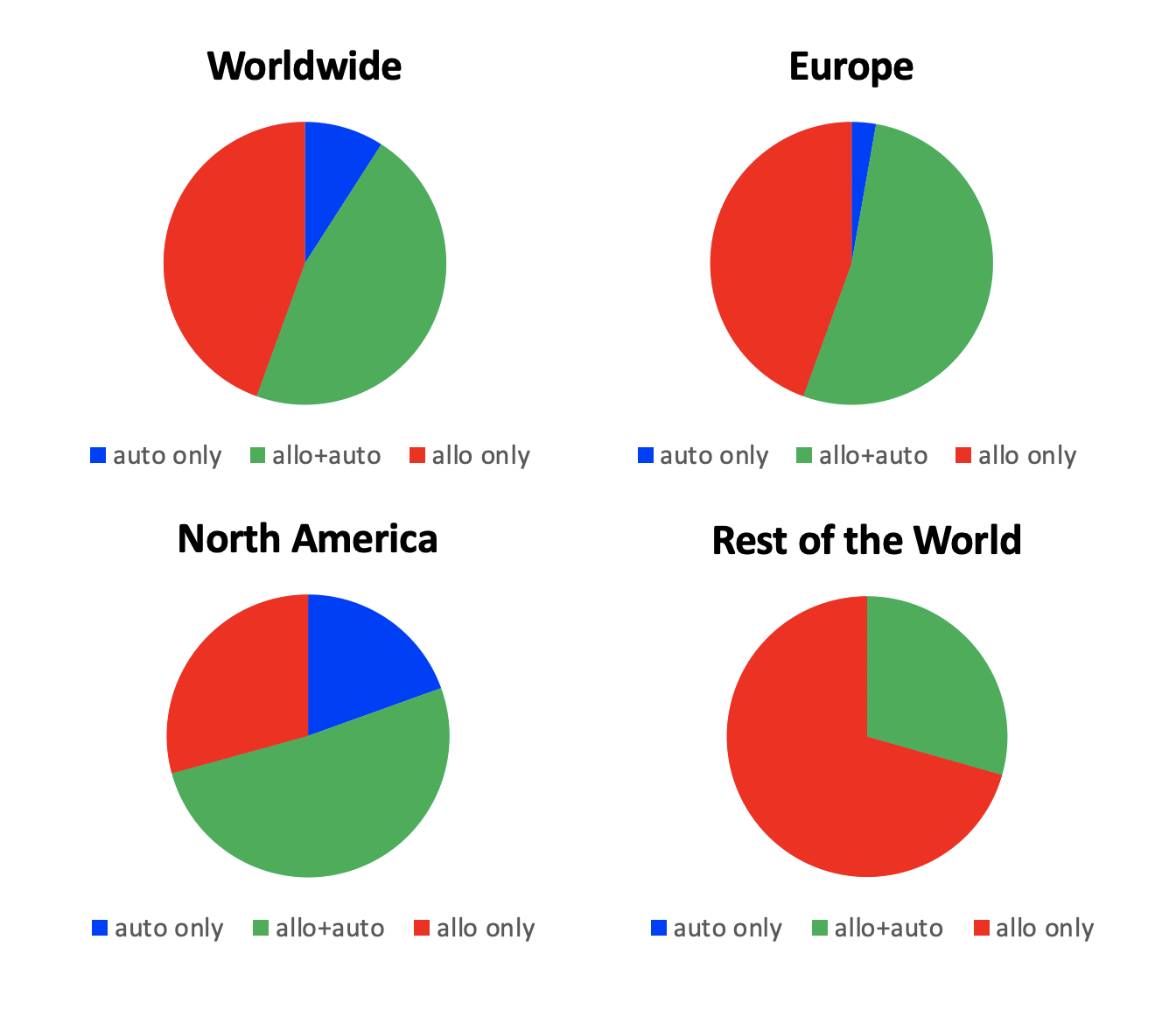

Supplement: Supplementary file 2 [file Image1.TIFF]

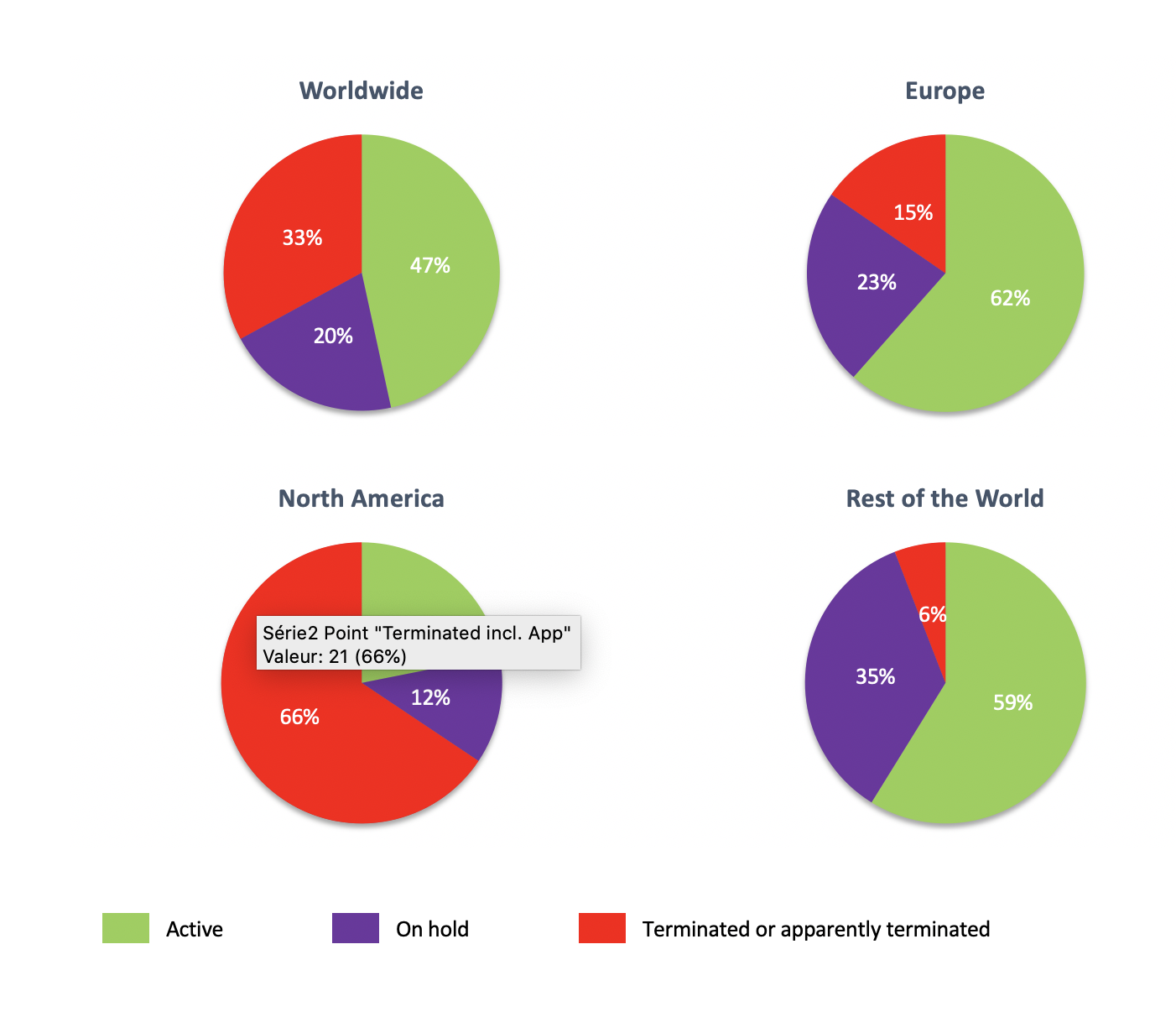

Supplement: Supplementary file 5 [file Image2.TIFF]
