# Supplementary material for: A Worldwide Survey of Activities and Practices in Clinical Islet of Langerhans Transplantation
Source: Transpl Int. 2022 Aug 11;35:10507. doi: 10.3389/ti.2022.10507 (PMC9402897; doi:10.3389/ti.2022.10507)
Supplement: Supplementary file 3 [file Table1.pdf]

Supplementary Table 1: List of islet transplant centers

Institution	City	Country	Continent	Invited to survey	Responded to survey	Source of activity data	First allogeneic islet transplant	Current status	Number of transplants	Number of patients
Changsha University of Science and	Changsha	China	Asia	Yes	No	2013 survey		unknown	16	12
Xiamen University	Fuzhou	China	Asia	Yes	No	Publication	2006	unknown	31	20
Shanghai Changzheng Hospital	Shanghai	China	Asia	Yes	No	none	n/a	unknown	unknown	unknown
Tianjin First Center Hospital	Tianjin	China	Asia	Yes	No	none	n/a	unknown	unknown	unknown
Tehran University of Medical Scienc	Tehran	Iran	Asia	Yes	Yes	Survey	2011	On hold	3	2
East National Hospital	Chiba	Japan	Asia	(yes)	(yes)	Survey	n/a	Active	8	n/a
Fukuoka University	Fukuoka	Japan	Asia	(yes)	(yes)	Survey	n/a	Active	4	n/a
Fukushima Prefectural University	Fukushima	Japan	Asia	(yes)	(yes)	Survey	n/a	Active	1	n/a
Japan Islet Transplant Consortium	***Japan Islet Transplant Co***	Japan	Asia	Yes	Yes	Survey	2004	Active	50	36
Kobe University	Kobe	Japan	Asia	(yes)	(yes)	Survey	n/a	Active	3	n/a
Kyoto University	Kyoto	Japan	Asia	Yes	Yes	Survey	2004	Active	28	13
Tohoku University	Sendai	Japan	Asia	(yes)	(yes)	Survey	n/a	Active	3	n/a
National Center for Global Health ar	Tokyo	Japan	Asia	Yes	Yes	Survey	2017	On hold	1	1
Catholic University of Korea	Seoul Catholic	Korea	Asia	Yes	Yes	Survey	2013	Active	4	3
Seoul National University Hospital	Seoul National	Korea	Asia	Yes	Yes	Survey	2012	On hold	1	1
Samsung Medical Center	Seoul Samsung	Korea	Asia	Yes	Yes	Survey	2000	On hold	30	10
Republican Center for Organ and Tis	Minsk	Belarus	Europe	Yes	Yes	Survey	2016	On hold	10	10
Louvain Catholic University	Bruxelles	Belgium	Europe	Yes	No	2013 survey	2002	Terminated	10	10
Brussels Free University	Bruxelles	Belgium	Europe	Yes	Yes	Survey	1994	Active	273	102
IKEM	Prague	Czech Republi	Europe	Yes	Yes	Survey	2005	Active	114	68
University of Helsinki	Helsinki	Finland	Europe	(yes)	(yes)	Survey	n/a	On hold	51	51
Université de Franche Comté	Besançon	France	Europe	(yes)	(yes)	Survey	1999	On hold	9	5
Université de Grenoble	Grenoble	France	Europe	(yes)	(yes)	Survey	1999	Active	79	41
Université de Lille	Lille	France	Europe	Yes	Yes	Survey	1998	Active	171	61
Hospices Civils de Lyon	Lyon	France	Europe	(yes)	(yes)	Survey	1999	Active	34	18
Université de Montpellier	Montpellier	France	Europe	(yes)	(yes)	Survey	2006	Active	52	22
Université de Lancy	Nancy	France	Europe	(yes)	(yes)	Survey	2008	On hold	7	3
Hopital Saint-Louis	Paris	France	Europe	Yes	Yes	Survey	2014	Active	23	10
Université de Strasbourg	Strasbourg	France	Europe	(yes)	(yes)	Survey	1999	Active	71	36
GRAGIL Consortium	***GRAGIL***	France/Switze	Europe	(yes)	(yes)	Survey	1999	Active	457	234
Technische Universität Dresden	Dresden	Germany	Europe	Yes	Yes	Survey	2008	Active	12	12
Justus Liebig University	Giessen	Germany	Europe	No	n/a	2013 survey	1992	Terminated	46	34
Semmelweis University	Budapest	Hungary	Europe	No	n/a	2013 survey	2003	Terminated	8	4
Niguarda Hospital	Milan	Italy	Europe	Yes	Yes	Survey	2009	Active	41	28
San Raffaele Institute	Milan	Italy	Europe	Yes	Yes	Survey	1989	Active	162	87
ISMETT	Palermo	Italy	Europe	Yes	Yes	Survey	2012	On hold	3	3
Oslo University	Oslo	Norway	Europe	Yes	Yes	Survey	2010	Active	127	48
Medical University Gdansk	Gdansk	Poland	Europe	Yes	Yes	Survey	2018	On hold	6	5
Foundation of Research and Science	Warsaw	Poland	Europe	Yes	Yes	Survey	2008	On hold	6	6
Nordic Network for Clinical islet tra	***Nordic Network***	Scandinavia	Europe	(yes)	(yes)	Survey	2001	Active	458	199
Hospital Clinic	Barcelone	Spain	Europe	Yes	Yes	Spanish ONT	2008	Terminated	3	2
University of Malaga	Malaga	Spain	Europe	Yes	Yes	Spanish ONT	2010	Terminated	3	2
Principe de Asturias University Hosp	Oviedo	Spain	Europe	Yes	Yes	Spanish ONT	2006	Terminated	1	1
Gothenburg University	Gothenburg	Sweden	Europe	(yes)	(yes)	Survey	n/a	Active	7	2
Malmö University Hospital	Malmö	Sweden	Europe	(yes)	(yes)	Survey	n/a	Active	37	12
Karolinska Institute	Stockholm	Sweden	Europe	Yes	Yes	Survey	2001	Active	69	26
Uppsala University	Uppsala	Sweden	Europe	(yes)	(yes)	Survey	2001	Active	167	60
Université de Genève	Genève	Switzerland	Europe	Yes	Yes	Survey	1994	Active	205	115
Universitätsspital Zurich	Zurich	Switzerland	Europe	Yes	Yes	Survey	2000	Active	120	54
Leiden University Medical Center	Leiden	The Netherlar	Europe	Yes	Yes	Survey	2007	Active	98	49
University of Bristol	Bristol	UK	Europe	(yes)	(yes)	Survey/NHS-BT	2012	On hold	4	3
Edinburgh Royal Infirmary	Edinburgh	UK	Europe	Yes	Yes	Survey	2011	Active	113	60
King's College	London	UK	Europe	Yes	Yes	Survey	2005	Active	27	13
Royal Free Hospital	London	UK	Europe	(yes)	(yes)	Survey/NHS-BT	2009	On hold	21	13
Manchester Royal Infirmary	Manchester	UK	Europe	Yes	Yes	Survey	2010	Active	36	24
Freeman Hospital	Newcastle-upon-Tyne	UK	Europe	Yes	Yes	Survey	2008	Active	56	32
University of Oxford	Oxford	UK	Europe	Yes	Yes	Survey	1992	Active	74	44
UK Islet Transplant Consortiun (UKI	***UK Consortium***	UK	Europe	(yes)	(yes)	Survey/NHS-BT	2008	Active	331	189
University of Alberta	Edmonton	Canada	North Amer	Yes	Yes	Survey	1989	Active	681	293
McGill University	Montreal	Canada	North Amer	Yes	Yes	Survey	2015	Active	9	6
Toronto General Hospital	Toronto	Canada	North Amer	No	n/a	CITR	2005	Apparently termi	1	1
University of British Columbia	Vancouver	Canada	North Amer	Yes	Yes	Survey	2003	Active	142	60
Emory University	Atlanta	USA	North Amer	Yes	No	CITR	2003	Apparently termi	19	12
University of Maryland	Baltimore	USA	North Amer	No	n/a	CITR	2002	Apparently termi	2	1
NIH Clinical Transplant Center	Bethesda	USA	North Amer	No	n/a	CITR	2000	Apparently termi	6	6
University of Alabama	Birmingham	USA	North Amer	No	n/a	CITR	2004	Apparently termi	3	1
Massachussets General Hospital	Boston	USA	North Amer	Yes	Yes	Survey	2000	On hold	17	9
Carolinas Medical Center	Charlotte	USA	North Amer	No	n/a	CITR	2003	Apparently termi	1	1
University of Virginia	Charlottesville	USA	North Amer	Yes	No	CITR	2004	unknown	6	2
University of Illinois	Chicago	USA	North Amer	Yes	No	CITR	2005	unknown	39	13
Northwestern University	Chicago	USA	North Amer	Yes	Yes	Survey	2003	Terminated	30	17
University of Chicago	Chicago	USA	North Amer	Yes	Yes	Survey	2005	Active	58	29
University of Cincinatti	Cincinatti	USA	North Amer	No	n/a	CITR	2001	Apparently termi	6	2
Ohio State University	Columbus	USA	North Amer	No	n/a	CITR	2011	Apparently termi	2	1
Baylor Simmons Transplant Institute	Dallas	USA	North Amer	Yes	Yes	Survey	2005	On hold	29	16
University of Colorado Health Scienc	Denver	USA	North Amer	No	n/a	CITR	2004	Apparently termi	5	2
City of Hope National Medical Cente	Duarte	USA	North Amer	Yes	Yes	Survey	2004	Active	61	31
Baylor Methodist Hospital	Houston	USA	North Amer	Yes	Yes	Survey	2003	Terminated	36	18
University of Wisconsin	Madison	USA	North Amer	Yes	Yes	Survey	2002	Terminated	25	11
University of Tennessee	Memphis	USA	North Amer	No	n/a	CITR	2002	Apparently termi	3	1
University of Miami	Miami	USA	North Amer	Yes	Yes	Survey	1985	On hold	100	56
University of Minnesota	Minneapolis	USA	North Amer	Yes	Yes	Survey	1974	Active	75	51
Columbia University	New York	USA	North Amer	Yes	No	CITR	2004	Apparently termi	1	1
Weill Cornell Medical College	New York	USA	North Amer	No	n/a	CITR	2004	Apparently termi	3	1
University of Pennsylvania	Philadelphia	USA	North Amer	Yes	Yes	Survey	2001	On hold	52	32
Mayo Clinic	Rochester	USA	North Amer	Yes	Yes	Survey	2008	Terminated	3	2
Washington University	Saint Louis	USA	North Amer	No	n/a	CITR	1989	Apparently termi	8	3
UCSF	San Francisco	USA	North Amer	Yes	Yes	Survey	2003	Active	40	30
Benaroya Research Institute	Seattle	USA	North Amer	No	n/a	CITR	2002	Apparently termi	4	2
Swedish Medical Center	Seattle	USA	North Amer	No	n/a	CITR	2002	Apparently termi	2	1
University of Washington	Seattle	USA	North Amer	No	n/a	CITR	2002	Apparently termi	1	1
University of Massachussets Memor	Worcester	USA	North Amer	No	n/a	CITR	2002	Apparently termi	5	2
Royal Adelaide Hospital	Adelaide	Australia	Oceania	Yes	Yes	Survey	2010	Active	22	11
Australian Islet Consortium	***Australian Islet Consortiu***	Australia	Oceania	(yes)	(yes)	Survey/ANZIPT	2012	Active	119	65
Saint Vincent institute	Melbourne	Australia	Oceania	Yes	Yes	Survey	2007	Active	41	21
Westmead Hospital	Sydney	Australia	Oceania	Yes	Yes	Survey/ANZIPT	2002	Active	56	33
Hospital Italiano	Buenos Aires	Argentina	South Amer	Yes	Yes	Survey	1995	On hold	18	16
University of Sao Paulo	Sao Paolo	Brazil	South Amer	Yes	Yes	Survey	2004	On hold	9	4
Fundacion Valle del Lilli	Cali	Colombia	South Amer	Yes	Yes	Personal conta	2014	Terminated	1	1
