# Supplementary material for: A Worldwide Survey of Activities and Practices in Clinical Islet of Langerhans Transplantation
Source: Transpl Int. 2022 Aug 11;35:10507. doi: 10.3389/ti.2022.10507 (PMC9402897; doi:10.3389/ti.2022.10507)
Supplement: Supplementary file 4 [file DataSheet1.pdf]

SURVEY ON ISLET ISOLATION AND TRANSPLANTATION ACTIVITY

...

* Required

1. Enter you Institution name *

Enter your answer

2. City *

Enter your answer

3. Country *

Enter your answer

4. What is the specialty of the islet transplant program principal investigator ? *

◯ Surgeon

◯ Endocrinologist

◯ Nephrologist

◯ Other

5. If you selected "Other", please specify

Enter your answer

6. What year was the first allogeneic islet transplant performed at your institution ? *

Enter your answer

7. As of 2021, what is the status of your islet ALLO-transplantation program ? *

- ◯ Active
- ◯ Temporarily on hold
- ◯ Terminated

8. What best describes your program ? *

- ◯ Islet isolation lab and islet transplantation program
- ◯ Islet isolation lab and local islet transplantation program
- ◯ Islet isolation lab and islet transplantation within network/consortium or bilateral collaboration
- ◯ Islet transplantation program only, as part of a bilateral collaboration
- ◯ Islet transplantation program only, as part of a network/consortium

9. If your institution works as part of a network or in bilateral collaborations, please indicate the name of your network and how many centers participate to it

Enter your answer

10. If your institution works as part of a network or in bilateral collaborations, please list the names of institutions participating to network or collaboration

Enter your answer

11. How many ALLOGENEIC islet transplant PROCEDURES have you performed at your institution SINCE 2000 ? *

Enter your answer

12. In how many different PATIENTS ? *

Enter your answer

13. How many allogeneic islet transplant procedures have you performed in your NETWORK since 2000 ?

Enter your answer

14. In how many different patients ?

Enter your answer

15. How many allogeneic islet transplant procedures have you performed at your institution IN 2020 ? *

Enter your answer

16. In how many different patients ? *

Enter your answer

17. How many allogeneic islet transplant procedures have you performed in your network in 2020 ?

Enter your answer

18. In how many different patients ?

Enter your answer

19. Which types of islet allo-transplantation procedures do you perform at your institution/network (more than one answer possible) ? *

- [ ] Islet Transplant Alone (ITA)
- [ ] Islet-after-Kidney (IAK)
- [ ] Simultaneous Islet-Kidney (SIK)
- [ ] Simultaneous Islet-Lung or Islet-after-Lung
- [ ] Simultaneous Islet-Liver
- [ ] Other combinations

20. If you selected "Other combinations", please specify

Enter your answer

21. Which type of islet allo-transplantation procedure is the preferred and most commonly performed at your institution/network (more than one answer possible) ? *

- ☐ ITA
- ☐ IAK
- ☐ SIK

22. Do you also perform islet auto-transplantation procedures at your institution ? *

- ◯ Yes
- ◯ No

23. If you answered yes, how many islet AUTOtransplantation procedures have you performed at your institution SINCE 2000?

Enter your answer

24. If you answered yes to question 22, how many islet AUTOtransplantation procedures have you performed at your institution IN 2020?

Enter your answer

25. What are the indications for islet auto-transplantation at your institution (more than one answer possible) ? *

- ☐ Chronic pancreatitis
- ☐ Benign tumors
- ☐ Malignant tumors
- ☐ Trauma
- ☐ Other

- [ ] Not performed

26. If you selected "Other", please specify

Enter your answer

27. What is the preferred and most common indication for islet auto-transplantation at your institution ? *

- ◯ Chronic pancreatitis
- ◯ Benign tumors
- ◯ Malignant tumors
- ◯ Other
- ◯ Not performed

28. If you selected "Other", please specify

Enter your answer

29. What types of donors are included in your islet allo-transplant program (more than one answer possible) ? *

- [ ] DBD
- [ ] DCD Maastricht II
- [ ] DCD Maastricht III, IV or V
- [ ] DCD Maastricht I

30. Have you performed CLINICAL islet auto- or allotransplantation in extrahepatic sites ? *

- ◯ Yes
- ◯ No

31. If you answered "yes", in what type of transplant ?

- ◯ allogeneic only
- ◯ autologous only
- ◯ both

32. If you answered "yes" to question 30, which sites have you used CLINICALLY ?

- ☐ Skeletal muscle
- ☐ Omentum
- ☐ Gastric submucosa
- ☐ Bone marrow
- ☐ Other
- ☐ Inside device

33. If you answered "other" or "inside device", please specify

Enter your answer

34. Was you islet transplant program interrupted at any time since 2000 ? *

- ◯ No

◯ Yes, for allotransplantation only

◯ Yes, for allo- and autotransplantation

◯ Yes for auto-transplantation only

35. If you answered "yes", is your islet transplantation program still interrupted ?

◯ Yes

◯ No

36. If you entered "yes" to question 29, please indicate the approximate duration and dates of interruption

Enter your answer

37. If you entered "yes" to question 29, please briefly indicate the reason

Enter your answer

38. What was the impact of the COVID-19 pandemic on your islet transplantation program (more than one answer possible) ? *

☐ No impact/maintained activity

☐ Decrease in isolation activity

☐ Decrease in allotransplantation activity

☐ Decrease in autotransplantation acitivity

☐ Interruption of allotransplantation program

☐ Interruption of autotransplantation program

39. Is vascularized pancreas transplantation also performed at your institution ? *

- Yes
- No

40. If vascularized pancreas transplantation is also performed at your institution, how are the islet and pancreas transplant programs connected)

- Single program (one program director)
- Two different programs with close interactions
- Two different programs with occasional interactions
- Two fully separated programs

41. If vascularized pancreas transplantation is also performed at your institution, are candidates for transplant first seen in a unique visit, or are they referred to separate visits?

- Unique
- Separate

42. If vascularized pancreas transplantation is also performed at your institution, are patients discussed in ... ?

- a single waiting list conference
- two separate waiting list conferences

43. In your country, are islet and pancreas transplant candidates inscribed on the same waiting list or on separate waiting lists? *

- Single waiting list

- ◯ Separate waiting lists

44. In your country, what is the status of islet isolation and transplantation reimbursement by the national health care system) *

- ◯ Reimbursed
- ◯ Process initiated for reimbursement approval
- ◯ Not reimbursed, but under evaluation
- ◯ Not reimbursed, no clear perspective

45. Which of the following components of your islet isolation and transplantation program are reimbursed by the national health care system (more than one answer possible) ? *

- ☐ Allo-transplantation, isolation
- ☐ Allo-transplantation, transplantation
- ☐ Allo-transplantation, follow-up
- ☐ Auto-transplantation, isolation
- ☐ Auto-transplantation, transplantation
- ☐ Auto-transplantation, follow-up
- ☐ None

46. How fully reimbursed is it ?

- ◯ fully
- ◯ partially
- ◯ not reimbursed

47. Do you have any additional comment about specificities of your islet allogeneic transplantation program ?

Enter your answer

Submit

Never give out your password. Report abuse

This content is created by the owner of the form. The data you submit will be sent to the form owner. Microsoft is not responsible for the privacy or security practices of its customers, including those of this form owner. Never give out your password.

Powered by Microsoft Forms |
The owner of this form has not provided a privacy statement as to how they will use your response data. Do not provide personal or sensitive information.
| Terms of use
